# Identification and Antibacterial Evaluation of Bioactive Compounds from *Garcinia kola* (Heckel) Seeds

**DOI:** 10.3390/molecules17066569

**Published:** 2012-05-31

**Authors:** Christinah T. Seanego, Roland N. Ndip

**Affiliations:** 1 Microbial Pathogenecity and Molecular Epidemiology Research Group, Department of Biochemistry and Microbiology, Faculty of Science and Agriculture, University of Fort Hare, Private Bag X 1314, Alice 5700, South Africa; Email: christinahseanego@yahoo.com; 2 Department of Microbiology and Parasitology, Faculty of Science, University of Buea, Box 63, Buea, Cameroon

**Keywords:** *Garcinia kola*, medicinal plants, antimicrobial activity, Minimum Inhibitory Concentration, Minimum Bactericidal Concentration, GC-MS

## Abstract

We assessed the bioactivity of *G. kola* seeds on *Streptococcus pyogenes*, *Staphylococcus aureus*, *Plesiomonas shigelloides* and *Salmonella typhimurium*. The crude ethyl acetate, ethanol, methanol, acetone and aqueous extracts were screened by the agar-well diffusion method and their activities were further determined by Minimum Inhibitory Concentration (MIC) and Minimum Bactericidal Concentration (MBC) assays. The extracts were fractionated by Thin Layer Chromatography (TLC). Bioautography was used to assess the activity of the possible classes of compounds present in the more active extracts. Column chromatography was used to purify the active compounds from the mixture, while GC-MS was used to identify the phytocomponents of the fractions. The inhibition zone diameters of the extracts ranged from 0–24 ± 1.1 mm, while MIC and MBC values ranged between 0.04–1.25 mg/mL and 0.081–2.5 mg/mL, respectively. The chloroform/ethyl acetate/formic acid (CEF) solvent system separated more active compounds. The MIC of the fractions ranged between 0.0006–2.5 mg/mL. CEF 3 (F3), CEF 11 (F11) and CEF 12 (F12) revealed the presence of high levels of linoleic acid, 1,2-benzenedicarboxylic acid and 2,3-dihydro-3,5-dihydroxy-6-methyl ester, respectively. The results obtained from this study justify the use of this plant in traditional medicine and provide leads which could be further exploited for the development of new and potent antimicrobials.

## 1. Introduction

The problem of antibacterial resistance to commonly used antibiotics coupled with the emergence of new and re-emerging diseases has led to a search for newer and alternative compounds for the treatment of drug-resistant infections [1]. Several findings on the chemotherapeutic potential of plants have shown that they can be sources of potent antimicrobial compounds [2,3]. *Streptococcus pyogenes* and *Staphylococcus aureus* are Gram-positive, non-spore forming, facultative anaerobic bacteria that are able to invade via the broken skin or mucous membrane. Infections caused by *S. pyogenes* include pharyngitis, localized skin infections, rheumatic fever, rheumatic heart disease and streptococcal toxic shock syndrome [4,5,6]; while *S. aureus* causes skin lesions such as boils, furuncules and more serious infections such as pneumonia, phlebitis, meningitis and urinary tract infections. These organisms have been recognized as having the ability to develop resistance to antibiotics [7,8,9,10]. *Plesiomonas shigelloides* and *Salmonella typhimurium* are Gram-negative, non-spore forming bacteria that are known to cause gastroenteritis with fever, chills, diarrhea, abdominal pain and vomiting [11,12]. The emergence of multidrug-resistant serotypes, especially *S. typhimurium* definitive phage-type (DT) 104, has become a potential problem [13] and therefore effective antimicrobials are essential for treatment.

*G. kola* is a medium sized evergreen tree which grows about 15–17 m high [14]. It is cultivated and distributed throughout West and Central Africa and found mostly in moist conditions. It produces a characteristic smooth elliptically shaped seeds, with yellow pulp and brown seed coat. It is also referred to as “bitter *kola”* because of the astringent taste. The seeds are culturally and socially significant in some parts of West Africa and are served for traditional hospitality in private, social and cultural functions. The seeds have been found to have broad spectrum antibacterial activity [15]. This has been demonstrated with methanolic extracts of the seeds on *Bacillus subtilis* (NCIB 3610), *Streptococcus faecalis* (NCIB 775), *Staphylococcus aureus* (NCIB 8588), *Klebsiella pneumoiae* (NCIB 418), *H. pylori* [15,16] amongst other organisms. Phytochemical compounds such as biflavonoids [17], biflavonones [18], triterpenes, xanthones and benzophenones [19] have been isolated from the seeds. Although studies have been done with this plant, solvents including water, petroleum, butanol and diethyl ether were used which might limit the antimicrobial potentials of plants, since the type of solvent used for plant extraction may have an effect on the nature of compounds extracted and the resulting bioactivity of the extract [20]. The overall objective of the present study was to ascertain the bioactivity of extracts and fractions of *Garcinia kola* seeds on selected bacterial pathogens; and identify the probable compounds present in the fractions.

## 2. Results and Discussion

### 2.1. Susceptibility Testing

The zones of inhibition (clear zones on agar) for all four organisms were measured in mm and the breakpoint for susceptibility was taken as ≥11 mm [16]. The water extract demonstrated no activity against any of the test organisms, whilst some extracts showed varying activity with inhibition zone diameters ranging from 0–24 ± 1.1 mm (Table 1). The methanol extract demonstrated a bigger zone diameter of 24 ± 1.1 mm for *S. pyogenes* (*p* < 0.05). Positive control zones ranged between 23–31 mm. The extract considered very active in this assay (methanol) was further evaluated to determine the Minimum Inhibitory Concentration (MIC).

**Table 1:** Zone of inhibition ± SD (mm) of the seed extracts of *G. kola* and ciprofloxacin (1.25) (mg/mL) against organisms.

	Zones of inhibition (mm) at different concentrations (mg/mL)				
Organism	Extract	50	100	200	Ciprofloxacin
	Ethyl Acetate	21 ± 1.3	23 ± 0.7	20 ± 1.2	25
	Acetone	19 ± 0.8	22 ± 2.3	23 ± 1.6	24
*S. pyogenes*	Ethanol	19 ± 0.6	19 ± 1.3	22 ± 0.9	24
	Methanol	20 ± 1.0	24 ± 1.1	21 ± 2.3	23
	Water	0	0	0	25
	Ethyl Acetate	17 ± 0.6	17 ± 1.6	14 ± 1.8	26
	Acetone	19 ± 1.6	19 ± 0.6	21 ± 1.1	24
*S. aureus*	Ethanol	18 ± 1.5	23 ± 0.8	20 ± 0.3	27
	Methanol	21 ± 1.1	22 ± 2.3	19 ± 1.3	23
	Water	0	0	0	24
	Ethyl Acetate	0	0	0	30
	Acetone	0	15 ± 1.3	12 ± 1.8	30
*P. shigelloides*	Ethanol	10 ± 2.3	19 ± 1.9	11 ± 2.3	31
	Methanol	18 ± 1.5	21 ± 1.3	19 ± 1.6	30
	Water	0	0	0	31
	Ethyl Acetate	0	0	0	30
	Acetone	16 ± 1.7	14 ± 2.3	0	30
*S. typhimurium*	Ethanol	0	18 ± 1.3	14 ± 1.8	29
	Methanol	17 ± 0.6	18 ± 1.3	15 ± 1.2	30
	Water	0	0	0	31

The results for susceptibility testing of the extracts which are indicated in Table 1 confirm the results of previous studies, which reported that methanol is an efficient solvent [21,22,23]. The water extract demonstrated poor activity in all the organisms since no zones of inhibition were seen on the agar plate; which is also in line with previous findings [20,24,25]. This is an indication that water was not a good solvent; probably because the compounds responsible for bioactivity were not soluble in distilled water. Our findings corroborates the fact that *S. aureus* has also been reported to be susceptible to *G. kola* extracts [1,15,21].

### 2.2. Minimum Inhibitory Concentration (MIC) and Minimum Bactericidal Concentration (MBC) Determination

The Minimum Inhibitory Concentration (MIC) and Minimum Bactericidal Concentration(MBC) were evaluated on the methanol extract that exhibited best activity against the test organisms. The MIC of the extract and ciprofloxacin ranged from 0.04–1.25 mg/mL and 0.0012–0.0195 mg/mL, respectively while the MBC of the extract and ciprofloxacin ranged from 0.081–2.5 mg/mL and 0.0781–0.3125 mg/mL, respectively (Table 2).

*S. pyogenes* and *S. aureus* had the lowest MIC of 0.04 mg/mL. The MBC values were higher than the MIC values. Thissuggests that the extract was bacteriostatic at lower concentration and bactericidal at higher concentration.Our observed susceptibility patterns for *S. pyogenes* and *S. aureus* may be due to the differences in thickness of the cell wall composition between Gram—negative and Gram—positive bacteria.

**Table 2:** Minimum Inhibitory Concentration (MIC_90)_ and Minimum Bactericidal Concentration (MBC) of methanol extracts of *G. kola* and ciprofloxacin on test organisms.

Organism	MIC values of Extract (mg/mL)	MIC values of Ciprofloxacin(mg/mL)	MBC values of Extracts(mg/mL)	MBC values of Ciprofloxacin (mg/mL)
*S. pyogenes*	0.04	0.0012	0.081	0.0781
*S. aureus*	0.04	0.0024	0.25	0.1563
*P. shigelloides*	1.25	0.0049	2.5	0.3125
*S. typhimurium*	0.63	0.0195	1.25	0.1563

### 2.3. Phytochemical Analysis

#### 2.3.1. Thin Layer Chromatography (TLC)

**Figure 1:**
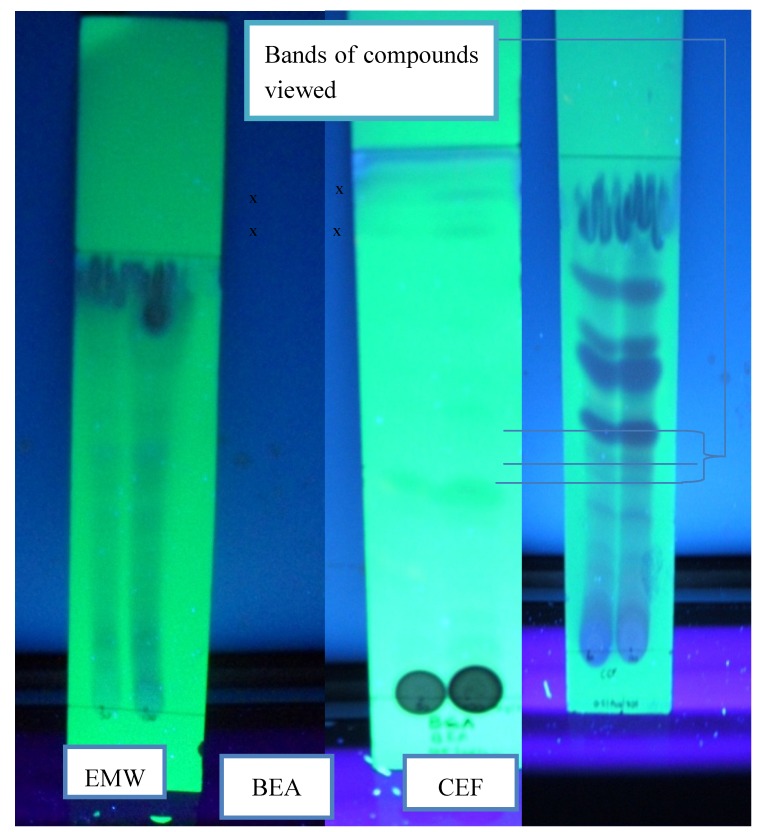
TLC plates showing separation of compounds using different solvent systems viewed under 302 nm (UV).

The three solvent combinations used were chloroform/ethyl acetate/formic acid [CEF] (10:8:2), ethyl acetate/methanol/water [EMW] (40:5.4:5) and benzene/ethanol/ammonium hydroxide [BEA] (18:2:0.2) at 50 and 100 mg/mL of methanol extract. CEF separated more bands, followed by EMW and BEA. Bands which were not seen on the TLC plates in daylight were visible when viewed under ultraviolet (UV) light at 302 nm (Figure 1). Most bands were visible in daylight with CEF plates while for BEA and EMW the bands were visible when viewed under 365 nm UV (figure not shown). In BEA, bands 3 and 6 showed the highest R*_f_* values of 0.12 and 0.32; band 1 showed lowest R*_f_* value of 0.08 and 0.01 at 50 mg/mL and 100 mg/mL, respectively. In CEF, bands 10 and 12 showed the highest R*_f_* values of 0.87 and 0.88 whereas band 1 showed lowest R*_f_* value of 0.09 and 0.08 at 50 mg/mL and 100 mg/mL, respectively. In EMW, bands 9 and 10 showed the highest R*_f_* values of 0.75 and 0.88 while band 1 showed the lowest R*_f_* value of 0.03 and 0.09 at 50 mg/mL and 100 mg/mL, respectively. A good separation was observed at a concentration of 100 mg/mL (Table 3).

Success in isolating compounds, which correspond to bands from the plant material, was largely dependent on the type of solvent combination used in the extraction process. BEA separated fewer compounds, with three bands at 50 mg/mL and six at 100 mg/mL, as opposed to CEF which had the highest separation. This implies that solvent with intermediary polarity separated more active compounds. This finding correlates that of Masoko [26], where the greatest separation was obtained using CEF.

**Table 3:** R*_f_* values for the different systems at different concentrations.

	Solvent systems						
Bands	BEA		CEF		EMW		
	**50**	**100**	**50**	**100**	**50**		**100**
1	0.08	0.01	0.09	0.08	0.03		0.09
2	0.1	0.13	0.14	0.11	0.08		0.16
3	0.12	0.16	0.39	0.19	0.11		0.19
4	-	0.22	0.5	0.39	0.16		0.31
5	-	0.28	0.52	0.53	0.31		0.49
6	-	0.32	0.53	0.55	0.49		0.56
7	-	-	0.63	0.63	0.56		0.64
8	-	-	0.72	0.64	0.72		0.67
9	-	-	0.83	0.72	0.75		0.76
10	-	-	0.87	0.83	-		0.88
11	-	-	-	0.85	-		-
12	-	-	-	0.88	-		-

-, No R_f_ values determined.

#### 2.3.2. Antimicrobial Activity Assay by Bioautography

The areas of inhibition (coloured white/light yellow on a purple/pink background) were compared with the R*_f_* of the related spot on the reference plate. In CEF, active compounds against *S. pyogenes* were found at varying R*_f_* values (0.53, 0.64), *S. aureus* (0.82), *P. shigelloides* (0.63, 0.72, 0.85) and *S. typhimurium* (0.53, 0.63, 0.55, 0.64) at 50 and 100 mg/mL, respectively. Compounds containing inhibitory potential in EMW for *S. pyogenes* were located at R*_f_* 0.30, 0.31, *S. aureus* at 0.56, 0.63, *P. shigelloides* at 0.75, 0.76, 0.88 and *S. typhimurium* at 0.52. In BEA, inhibitory compounds were found at the origin for both *S. pyogenes* and *S. typhimurium* only. Most compounds having inhibitory effect were found in CEF chromatograms, followed by EMW and lastly BEA (Table 4). 

**Table 4:** Inhibition of bacterial species by methanol extract using bioautography.

Organisms	a		b		Solvent system
	(50 mg/mL	100 mg/mL)	(50 mg/mL	100 mg/mL)	
*S. pyogenes*	0.53	0.53	+++	+++	CEF
	0.64	0.64	+++	+++	CEF
	0.30	0.31	+	+	EMW
	origin	Origin	++++	++++	BEA
*S. aureus*	-	0.53	-	+	CEF
	0.56	0.63	+++	+++	EMW
	-	-	-	-	BEA
*P. shigelloides*	0.63	0.63	++	++	CEF
	0.73	0.72	++	+++	CEF
	-	0.85	-	++	EMW
	0.75	0.76	++	++	EMW
	-	0.88	-	++	EMW
	-	-	-	-	BEA
*S. typhimurium*	0.53	0.55	++	++	CEF
	0.63	0.64	++++	+++	CEF
	0.52	0.52	++	+++	EMW
	origin	Origin	++	++	BEA

a, Component R*_f_*, b, Degree of inhibition; *R_f_*, Ratio of the distance travelled by compound to the distance travelled by solvent up plate; -, Component not active; +, slight inhibition; ++, moderate inhibition; +++, high inhibition; ++++, very high inhibition; origin, spot on the TLC plate where the extract was initially applied.

In some cases no inhibition of microbial growth was observed on some parts of the plate, in line with previously published results [27]. The absence of activity was interpreted to be due to the evaporation of active compounds or presence of very little amount of active compounds during the removal of eluents [28]. It might also be due to traces of some solvents left in the chromatograms interfering with the compounds. Another explanation for the observed non-activity could be due to very weak activity of the extracts against the selected microorganisms. 

### 2.4. Column Chromatography Analysis and MIC90 Determination of Fractions

The solvent system that exhibited the best separation of compounds (CEF) was chosen for column chromatography. CEF separated 12 compounds at 100 mg/mL concentration more than EMW and CEF solvent combinations. Most fractions collected in this study were colourless. TLC fractions 4–6, 8–10 and 14–16 indicated similar compounds, and were combined to yield 16 compounds (Table 5). Eluted compound fractions were assayed for MIC_90_. MIC ranged between 0.0006–2.5 mg/mL and that of ciprofloxacin between 0.0012–0.0781 mg/mL (Table 5). The lowest MIC (0.0012 mg/mL) of CEF 18 (F18) against *S. pyogenes* compared favourably to that of ciprofloxacin (*p* < 0.05). This result may indicate that CEF 18 (F18) probably has the same inhibitory potential with ciprofloxacin (positive control). Most high MIC values of 2.5 mg/mL were observed against Gram-negative bacteria (*S. typhimurium* and *P. shigelloides*); this may be related to the thicker cell wall composition of Gram - negatives. Poor activity of some fractions may be due to insufficient amount of active ingredients.

**Table 5:** R*_f_* values of fractionated compounds and MICs in mg/mL against test organisms.

Fraction	R*f* value of fractionated compounds	MIC_90_ against test organisms			
		*S. pyogenes*	*S. aureus*	*P. shigelloides*	*S. typhimurium*
C 1	0.008, 0.159, 0.31	ND	ND	ND	ND
C2	0.09, 0.12, 0.36	ND	ND	ND	ND
C3	0.13, 0.17, 0.29	0.0195	0.00781	ND	ND
C4	0.156, 0.286, 0.294	0.00871	0.0195	ND	ND
CEF 1	0.2, 0.366, 0.42	0.049	0.0098	0.625	0.3125
CEF 2	0.34, 0.4, 0.52	0.625	0.024	0.625	1.25
CEF 3	0.153	0.1563	0.0781	0.0781	0.1563
CEF 4-6	0.13, 0.226, 0.3	ND	0.024	2.5	2.5
CEF 7	0.2, 0.33	0.625	0.0049	ND	0.1563
CEF 8-10	0.16, 0.306, 0.36	0.0195	0.024	2.5	2.5
CEF 11	0.13	0.049	0.0006	2.5	0.012
CEF 12	0.15, 0.18	0.3125	0.635	0.0781	1.25
CEF 13	0.13, 0.18, 0.28	0.0049	0.024	1.25	ND
CEF 14-16	0.15, 0.2	0.3125	1.25	1.625	ND
CEF 17	0.11, 0.38	0.0024	0.049	0.325	ND
CEF 18	0.12, 0.27	0.0012	0.1563	0.0195	ND
Ciprofloxacin		0.0012	0.0049	0.012	0.0781

C, Chloroform; CEF, (Chloroform: Ethyl Acetate: Formic acid); ND, Not Determined (value not within susceptible range).

### 2.5. Gas-Chromatography/Mass-Spectrometry (GC-MS)

**Figure 2:**
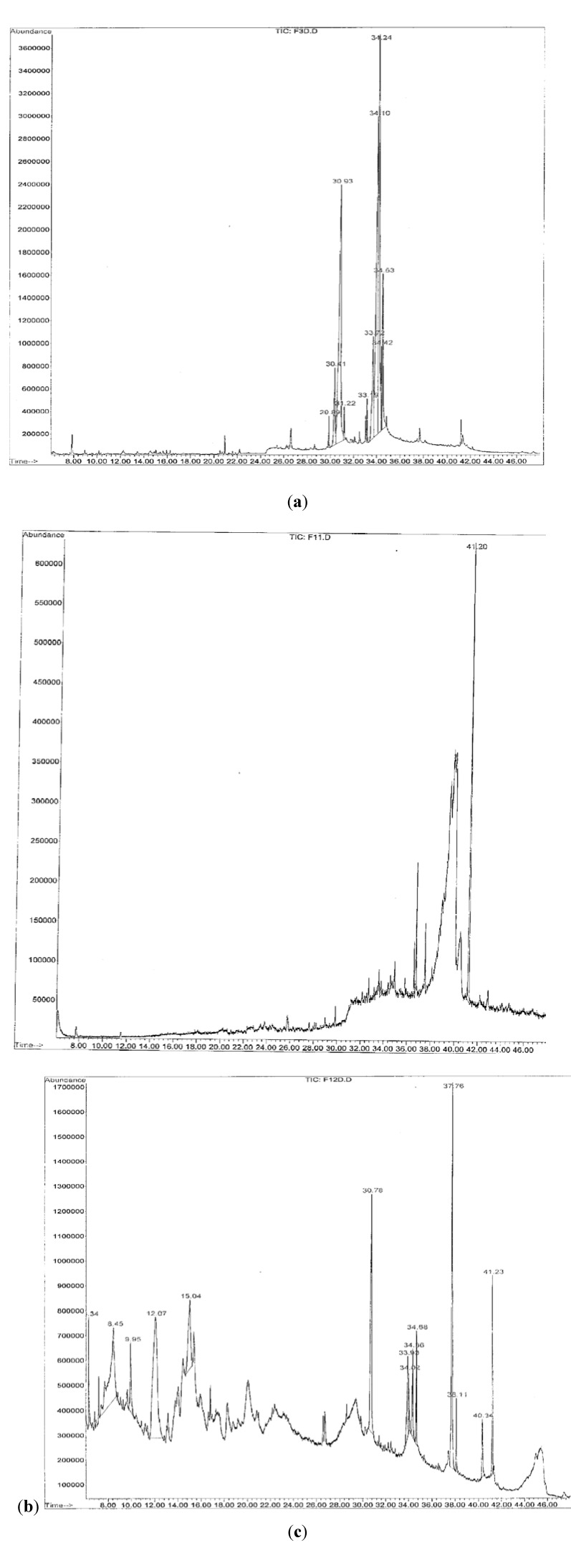
(**a**) GC-MS chromatogram of F3; (**b**) GC/MS chromatogram of F11; (**c**) GC-MS chromatogram of F12.

Three fractions CEF 3 (F3), CEF 11 (F11), and CEF 12 (F12) were analysed by GC-MS to determine the type(s) of compounds present. Figure 2(a–c) show chromatograms which are plots of the total mass eluting from GC and detected by MS as a function of time. Each peak represents a discrete chemical compound. These fractions were selected based on their purity [less bands shown by TLC profile (≤2 bands)] and their activity on the organisms. F3 and F11 showed one band on the TLC plate indicating better purity of the compounds, while F12 showed two bands. Although CEF 7, CEF 14-16, CEF 17 and CEF 18 indicated two bands on the TLC plate, CEF 7 did not show inhibitory activity on *P. shigelloides* and CEF 14-16, CEF 17, CEF 18 on *S. typhimurium*; they were therefore not analyzed by GC-MS. CEF 3 (F3) showed high level of linoleic acid (26.60%), followed by hexadecanoic acid (25.07%) and 9-octadecenoic acid (24.81%); 10 peaks were identified which indicate the likely presence of 10 compounds. F11 presented several peaks; it however, had the compound 1, 2-benzenedicarboxylic acid (100%) identified as the major peak from this fraction. F12 showed as major compounds 2,3-dihydro-3,5-dihydroxy-6-methyl ester (24.16%), followed by 1-butanol (15.72%) and 9-octadecenamide (13.82%) and 13 peaks were identified on the GC-MS chromatogram. 

Among other chemical compounds detected by GC-MS were 3,4,8-trimethyl-2-nonenal (2.07%), hexadecanamide (1.59%), and *n*-tetradecanoic acid amide (2.55%), though small quantities of these compounds were identified (Table 6). The compound 3,4,8-trimethyl-2-nonenal is probably new since no reports exist on it in the literature. Most compounds revealed by GC-MS in this study were fatty acids and have been reported to have antibacterial and antifungal activity [29,30,31,32,33]. The observed potent antimicrobial properties of the methanolic extract and fractions observed in our study could be likened to the presence of 1,2-Benzenedicarboxylic acid (100%), linoleic acid (26.60%), hexadecanoic acid, palmitic (25.07%), 2,3-dihydro-3,5-dihydroxy-6-methyl ester (24.16%), and 9-octadecadienoic acid (24.81%) since these volatile compounds where the major constituents and had high percentages.

**Table 6:** Phytochemicals of the CEF fractions of *Garcinia kola* seeds.

Fraction	Peak numbers	RT (mins)	Compounds identified	%
	1	29.89	Hexadecanoic acid, methyl ester	0.73
	2	30.42	Hexadecanoic acid	4.41
	3	31.93	Hexadecanoic acid, Palmitic acid	25.07
	4	31.22	Hexadecanoic acid, ethyl ester	0.83
CEF 3 / F3	5	33.19	9-Octadecenoic acid, methyl ester	0.91
	6	33.71	Heptadecene-(8)-carbonic acid	6.63
	7	34.10	Linoloic acid	26.60
	8	34.24	9-Octadecenoic acid	24.81
	9	34.42	14-Pentadecanoic acid	2.62
	10	41.20	Octadenoic acid, Stearic acid	7.49
**Total**				**100**
CEF 11 / F11	1	41.20	1.2-Benzenedicarboxylic acid	**100**
	1	6.63	Formamide, *N,N*-Diethyl	2.31
	2	8.45	1-Butanol	15.72
	3	9.95	3-Isothiazolecarboxamide	3.03
	4	12.07	2.3-Dihydro-3,5-dihydeoxy-6-methyl ester	24.16
	5	15.04	2.5-Di(hydroxymethyl)-furan	7.06
	6	30.78	Palmitic acid	12.91
CEF 12 / F12	7	33.94	9-Octadecanoic acid	4.08
	8	34.02	Cyclohexadecane	1.84
	9	34.36	Stearic acid	3.11
	10	34.68	*n*-Tetradecanoic acid amide	2.55
	11	37.75	9-Octadecenamide	13.82
	12	38.11	Hexadecanamide	1.59
	13	40.34	3, 4,8-Trimethyl-2-nonenal	2.07
**Total**				**94.24**

RT, Retention time; CEF, Chloroform/ethyl acetate/formic acid; F, Fraction.

## 3. Experimental

### 3.1. Bacterial Strains

Standard strains of the following organisms were used in this study. They included: *Streptococcus pyogenes* ATCC 49399, *Staphylococcus aureus* NCTC 6571, *Plesiomonas shigelloides* ATCC 51903 and *Salmonella typhimurium* ATCC 13311. These organisms are human pathogens and are leading causes of hospital and acquired infections. They were selected based on their prevalence and increasing trend of resistance to antibiotics [34,35,36].

### 3.2. Resuscitation of Bacterial Strains

Bacterial isolates of test organisms were obtained from stock cultures maintained at our laboratory. Resuscitation of the cultures was done by inoculating the organisms on Mueller Hinton, MacConkey and *Salmonella - Shigella* agar and plates incubated at 37 °C for 24 hours. They were frequently subcultured on Mueller Hinton (MHA) or Nutrient agar slants and stored in the refrigerator.

### 3.3. Preparation of Plant Extract

*Garcinia kola* seeds were purchased from a local market in Cameroon. They were blended into powder and kept in air-tight container for further use. The extracts of the seeds were prepared in accordance with the method of Basri and Fan [37]. Briefly, one hundred grams of the powdered seeds were steeped in 500 mL of ethyl acetate, ethanol, methanol, acetone and distilled water for 24 h with shaking (Orbital Incubator Shaker, Gallenkamp) at 140 rev/min. The resulting extracts were filtered using Whatman No 1 Filter paper. The extracts were further concentrated to dryness under reduced pressure at 37 °C using a rotary evaporator (Strike 202 Steroglass, Italy) to remove the solvents.

### 3.4. Antimicrobial Susceptibility Testing

Sensitivity testing of *G. kola* seeds extract was done using the agar well diffusion method as previously described by Irobi *et al.* [38] with minor modifications. The bacterial isolateswere grown in MHA. Plates were swabbed with cotton wool impregnated with the organisms prepared at 0.5 McFarland standard. Wells were bored into the agar medium using sterile 6 mm cork borer. Five holes were bored in one plate. The first three wells were filled with solution of the extract at concentrations of 200, 100 and 50 mg/mL. The other two wells were filled with a positive control (ciprofloxacin 1.25 mg/mL) and negative control [dimethylsulfoxide (DMSO)]. The plates were then allowed to stand for 20 min to allow proper diffusion of the solution into the medium before incubation. They were then incubated at 37 °C for 24 h. Antimicrobial activity was evaluated by measuring the zones of inhibition against the test organisms. The experiment was replicated two times and zones of inhibition reported as mean ± SD.

### 3.5. Determination of Minimum Inhibitory Concentration (MIC90)

The microdilution method was employed to determine the Minimum Inhibitory Concentration(MIC) of the plant extract (methanol) that gave the best antimicrobial activity using 96 well microtitre plates as previously described by Njume *et al.* [39]. A twofold serial dilution was carried out. Two to three colonies of the test organisms were grown in MH broth. Approximately 20 µL of each bacterial suspension was added to 180 µL of wells containing extract. Control wells were prepared by adding 100 µL of ciprofloxacin at a concentration of 1.25 mg/mL. The plates were incubated overnight at 37 °C and read with ELISA microplate reader (Model 680, Bio-Rad, Japan). The MIC_90_ was taken as lowest concentration of the test extract resulting in inhibition of 90% of bacterial growth.

### 3.6. Determination of Minimum Bactericidal Concentration (MBC)

The MBC was determined using the method of Vila *et al.* [40] with small modifications. Approximately, 2 µL of the sample from Minimum Inhibitory Concentration assay was spread onto freshly prepared MHA plates, incubated at 37 °C for 24 hours and monitored for the presence of bacterial growth. The MBC were taken as the lowest concentration that did not allow bacterial growth on the surface of the agar plates.

### 3.7. Phytochemical and Antimicrobial Analysis

#### 3.7.1. Thin Layer Chromatography (TLC)

Thin Layer Chromatography was used to analyse the chemical constituents of the *G.kola* seeds extracts using aluminium-backed TLC plates (Merck, silica gel 60 F254) according to the method described by Njume *et al.* [39]. Plates were spotted with 50 and 100 mg/mL of methanol extract of *G. kola* seeds. The TLC plates were developed with three eluent systems: EMW (40:5.4:5): CEF (10:8:2) and BEA (18:2:0.2). Development of the chromatograms was done in a closed TLC chamber containing solvent mixture and shaken. The plates were sprayed with vanillin solution (0.2 g vanillin + 28 mL of methanol + 1 mL of sulphuric acid) and allowed to dry for 5 min; the chromatograms were heated at 100 °C and allowed for optimal colour development. The spots or bands were visualized in broad daylight and also under ultraviolet light at 302 and 365 nm. The following formula was used to measure the retention factor (R*_f_*) which is distance the compound travels to the distance the solvent travels.


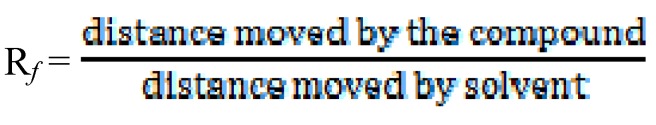


#### 3.7.2. Antimicrobial Activity Assay by Bioautography

The method developed by Masoko and Eloff [28] was used to determine active compounds. The plates developed in the three different mobile systems used: CEF, BEA and EMW were dried for up to a week at room temperature under a stream of air to remove the remaining solvent. The plates were sprayed with concentrated bacterial cultures and incubated in a humidified container at 37 °C overnight. The following day the plates were sprayed with 0.2 mg/mL solution of *p*-iodonitrotetrazolium violet (INT) (Sigma®, Aldrich South Africa). Purple/pink colour indicated an area were the organism grew and clear zones indicated the absence of the organism due to the presence of compound(s) that inhibited the growth of tested microorganisms. *R_f_* of the zones on the plates were compared with that on the reference plates to find the R*_f_* of the active compound.

### 3.8. Column Chromatography

Column chromatography was used as a purification technique. The mixture of compounds to be purified was dissolved in small amount of the appropriate solvent as earlier described [29]. A 40 cm long × 2.5 cm diameter glass column was packed to a height of 31 cm with a slurry of silica gel 60; particle size 0.063–0.2 mm/ 70–230 mesh (Merck, Germany). The mixture was then loaded onto a silica gel column equilibrated first with chloroform. The combination which gave good activity, CEF (10:8:2) was then used to elute the column; fractions (200 mL) were collected in bottles and coded C for fractions collected in chloroform and CEF for chloroform/ethyl acetate/formic acid. They were concentrated on a rotary evaporator (Strike 202 Steroglass, Italy) to remove excess solvents at a reduced pressure. 

### 3.9. Determination of Minimum Inhibitory Concentration (MIC90) of Fractions

The Minimum Inhibitory Concentration (MIC_90_) of the fractions was determined by the micro-broth dilution method performed in 96-well plate as previously described for the extracts [39]; active fractions were further analyzed on TLC to deternine the purity. Control wells were prepared with culture medium only and bacterial suspension plus broth. Ciprofloxacin was used as a positive control at 1.25 mg/mL. An automatic ELISA microplate reader (SynergyMx, Biotek^R^, USA) adjusted to 620 nm was used to measure the absorbance of the plates before and after incubation at 37 °C. The absorbencies were compared to detect an increase or deacrease in bacterial growth. The lowest concentration of the fraction resulting in inhibition of 90% bacterial growth was recorded as the MIC_90_.

### 3.10. Gas-Chromatography/Mass-Spectrometry (GC-MS)

The chemical constituents of fractions were analysed by GC/MS using a Hewlett-Packard HP 5973 mass spectrometer interfaced with an HP-6890 gas chromatograph equipped with an HP5 column (30 m × 0.25 mm i.d, 0.25 µm film thickness) and MS detector. Helium was used as a carrier gas (1 mi/min), a split ratio of 1:25 and scan range of 35 to 425 amu. The oven temperature was set from 70 °C (after 2 min) to 325 °C at 4 °C per minute and final temperature held for 10 min at 240 °C. The samples were injected into the GC-MS inlet port using a syringe. The ion source was set at 250 °C and electron ionization at 70 Ev. The compounds were identified based on the match with their mass spectra and retention indices with those of the Wiley 275 library (Wiley, New York) in the computer library and literature [41,42]. 

### 3.11. Statistical Analysis

Analysis was performed using SPSS version 18.0 (Chicago, IL, USA, 2009). The one way ANOVA test was used to determine if there was any statistically significant difference in the diameter of zones of inhibition of the plant extracts and ciprofloxacin; the MIC of the most active extract (methanol), fractions and positive control (ciprofloxacin). P values < 0.05 were considered significant.

## 4. Conclusions

The methanol extract and fractions of *Garcinia kola* seeds show promise as a new source of antibacterial compounds. Though 1,2-benzenedicarboxylic acid (100%), linoleic acid (26.60%), hexadecanoic acid (25.07%), 2,3-dihydro-3,5-dihydroxy-6-methyl ester (24.16%) and 9-octadecadienoic acid (24.81%) were identified as the major chemical compounds, further studies on their toxicity, *in vivo* potency and mechanism of action would be required to elucidate their potential usefulness. 

## References

[1] Sibanda T., Okoh A.I. (2008). *In vitro* antimicrobial regimes of crude aqueous and acetone extract of *Garcinia kola* seeds. J. Biol. Sci..

[2] Rabe T., Van Staden J. (1997). Antibacterial activity of South African plants used for medicinal purposes. J. Ethnopharmacol..

[3] Njume C., Afolayan A.J., Ndip R.N. (2011). Preliminary phytochemical screening and *in vitro* anti-*Helicobacter pylori* activity of acetone and aqueous extracts of the stem bark of *Sclerocaryabirrea* (Anacardiaceae). Arch. Med. Res..

[4] Stevens D.L. (1995). Streptoccoccal toxic shock syndrome spectrum of disease pathogenesis and new concepts in treatment. Emerging Infect. Dis..

[5] Ryan K.J., Ray G. (2004). Introduction to Infectious Diseases. Sherris Medical Microbiology.

[6] Smeersters P.R., Dreze P.A., Biarant D., Van Melderen L., Vergison A. (2010). Group A *Streptococcus* virulence and host factors in two toddlers with rheumatic fever following toxic shock syndrome. Int. J. Infect. Dis..

[7] Shibl A. (2005). Pattern of macrolide resistance determinants among *Streptoccocus pyogenes* and *Streptococcus pneumoniae* isolates in Saudi Arabia. J. Int. Med. Res..

[8] Oliveira D.C., Miheirico C., de Lencastre H. (2006). Redefining structural variant of staphylococcal cassette chromosome *mec*, SCC *mec* type VI. Antimicrob. Agents Chemother..

[9] Richter S.S., Heilmann K.P., Dohrn C.L., Beekmann S.E., Riahi F., García-de-Lomas J., Ferech M., Goossens H., Doern G.V. (2008). Increasing telithromycin resistance among *Streptococcus pyogenes* in Europe. J. Antimicrob. Chemother..

[10] Yan J., Wu H., Whang A., Fu H., Lee C., Wu T. (2000). Prevalence of polyclonal *mef-A* containing isolates among erythromycin resistant group A Streptococci in Southern Taiwan. J. Clin. Microbiol..

[11] Ueda Y., Suzuki N., Furukawa T., Takegaki Y., Takahashi N., Miyagi K. (1999). Analysis of enteropathogenic bacteria at Kansai Airport Quarantine Station from September 4th, 1994 through December 1996. Kansenshogaku Zasshi.

[12] McClelland M., Sanderson K.E., Spieth J., Clifton S.W., Latreille P., Courtney L., Porwollik S., Ali J., Dante M., Du F. (2001). Complete genome sequence of *Salmonella enterica* serovarTyphimurium LT2. Nature.

[13] Butaye P., Michael G.B., Schwarz S., Barrett T.J., Brisabois A., While D.G. (2006). The clonal spread of multidrug-resistant non-typhi *Salmonella* serotypes. Microb. Infect..

[14] Plowder C.C. (1972). A Manual of Plants Names.

[15] Adeboye M.F., Akinpelu D.A., Okoh A.I. (2008). The bioactive and phytochemical properties of *Garcinia kola* Heckel seed extract on some pathogens. Afr. J. Biotechnol..

[16] Njume C., Afolayan A.J., Clarke A.M., Ndip R.N. (2011). Crude ethanolic extracts of *Garcinia kola* seeds Heckel prolong the lag phase of *Helicobacter pylori*: inhibitory and bactericidal potential. J. Med. Food.

[17] Okunji C.O., Tantalia A.W., Hicks R.P., Iwu M.M., Skanchy D.J. (2002). Capillary electrophoresis determination of biflavonones from *Garcinia kola* in the traditional African medicinal formulations. Plant Med..

[18] Cotterhill P.J., Scheinmann F., Stenhouse T.A. (1978). Extractives from *Guttiferae*: Kolaflavanone, a new biflavanone from the nuts of *Garcinia kola* Heckel. J. Chem. Soc. Perkin Trans. 1.

[19] Okunji C., Komarnytsky S., Fear G., Poulev A., Ribnicky D.M., Awachie P.I., Raskinn I. (1151). Preparative isolation and identification of tyrosinase inhibitors from the seeds *of Garcinia kola* by high - speed counter - current chromatography. J. Chromatog..

[20] Eloff J.N. (1998). Which extractant should be used for the screening and isolation of antimicrobial component from plants?. J. Ethnopharmacol..

[21] Ezeifeka G.O., Onji M.U., Mbata T.I., Patrick A.O. (2004). Antimicrobial activities of *Cajanus Cajan*, *Garcinia kola* seeds and *Xylopia aethiopica* on pathogenic microorganisms.. J. Biol. Sci..

[22] Jayalakshmi B., Raveesha K.A., Amrutheth K.N. (2011). Phytochemical investigations and antibacterial activity of some medicinal plants against pathogenic bacteria. J. App. Pharm. Sci..

[23] Vaghasiya Y., Batel H., Chanda S. (2011). Antibacterial activity of *Mangifera inica* L seeds against some human pathogenic bacterial strains. Afr. J. Biotechnol..

[24] Manetti A.G., Zingaretti C., Falugi F., Capo S., Bombaci M., Bagnoli F., Gambellini G., Bensi G., Mora M., Edwards A.M. (2007). *Streptococcus pyogenes* pili promote pharyngeal cell adhesion and biofilm formation.. Mol. Microbiol..

[25] Nwaokorie C.F., Akitoye C., Folasade O., Gaetti-Jardim E., Oyedele G., Ayanbadejo P., Abdurrazaq T., Umezudike A. (2010). Antimicrobial activity of * Garcinia kola* on oral *Fusobacterium nucleatum* and biofilm. Afr. J. Microbiol. Res..

[26] Masoko P., Picard J., Eloff J.N. (2007). The antifungal activity of twenty-four Southern African *Combretum* species (Combretaceae). S. Afr. J. Bot..

[27] Masoko P., Eloff J.N. (2005). The diversity of antifungal compounds of six SA *terminalia* species *Combretaceae* determined by bioautography. Afr. J. Biotechnol..

[28] Masoko P., Mokgotho M.P., Mbazima V.G., Mampuru L.J. (2008). Biological activities of *Typhacapensis* (Typhaceae) from Limpopo province South Africa. Afr. J. Biotechnol..

[29] McGraw L.J., Jager A.K., Van Staden J. (2002). Isolation of antibacterial fatty acids from * Schotia brachybetala*. Fitoterapia.

[30] Seidel V., Tailor P.W. (2004). *In vitro* activity of extracts and constituents of Pelagonium against rapidly growing mycobacteria. Int. J. Antimicrob. Agents.

[31] Russel A.D. (1991). Mechanisms of bacterial resistance of non-antibiotics: Food additives and food pharmaceutical preservatives. J. Appl. Bacteriol..

[32] Eliyinmi A.F., Bressler D.C., Isiaka A., Sporns P., Oshodi A. (2006). Chemical composition of *Garcinia kola* seed and hulls. Pol. J. Food. Nutr. Sci..

[33] Gopalakrishan S., Saroja K., Dulcy E. (2011). GC-MS analysis of methanolic extract of leaves of *Dipleracanthus patulus*. J. Chem. Pharm. Res..

[34] Eloff J.N., Famakin J.O., Katerere D.R.P. (2005). Isolation of an antibacterial stilbene from *Combretum woodii* leaves. Afr. J. Biotechnol..

[35] Nethathe B.B., Ndip R.N. (2011). Bioactivity of *Hydonora africana* on selected bacterial pathogens: Preliminary phytochemical screening. Afr. J. Microbiol. Res..

[36] Nyenje M., Ndip R.N. (2011). *In-vitro* antimicrobial activity of the crude acetone extract of the stem bark of *Combretum molle* against selected bacterial pathogens of medical importance. J. Med. Plts. Res..

[37] Basri D.F., Fan S.H. (2005). The potential of aqueous and acetone extracts of gall of *Queercus infectoria* as antibacterial agents. Indian J. Pham. Sci..

[38] Irobi O.N., Moo-Young M., Anderson W.A., Daramola S.O. (1994). Antimicrobial activity of bark extracts of *Bridelia ferruginea* (Euphorbiaceae). J. Ethnopharmacol..

[39] Njume C., Afolayan A.J., Green E., Ndip R.N. (2011). Volatile compounds in the stem bark of *Sclerocarya birrea* possess antimicrobial activity against drug resistant strains of *Helicobacter pylori*. Int. J. Antimicrob. Agents.

[40] Vila R., Santana A.I., Peez-Roses R., Valderrama A., Castelli M.V., Mendonca S., Zacchino S., Gapta M.P., Salvador B.A. (2010). Composition and biological activity of the essential oil from leaves of *Plinia cerrocamanensis*, a new source of α-bisabolol. Bioresour. Technol..

[41] Adams P.R. (1989). Identification of Essential Oil Components by Ion Trap Mass Spectroscopy.

[42] Joulain D., Koenig W.A. (1998). The Atlas of Spectral Data of Sesquiterpine Hydrocarbons.

